# Editorial: Community series in mental illness, culture, and society: dealing with the COVID-19 pandemic, volume VII

**DOI:** 10.3389/fpsyt.2023.1247118

**Published:** 2023-07-12

**Authors:** Samer El Hayek, Renato de Filippis, Mohammadreza Shalbafan

**Affiliations:** ^1^Medical Department, Erada Center for Treatment and Rehab in Dubai, Dubai, United Arab Emirates; ^2^Psychiatry Unit, Department of Health Sciences, University Magna Graecia of Catanzaro, Catanzaro, Italy; ^3^Mental Health Research Center, Psychosocial Health Research Institute, Department of Psychiatry, School of Medicine, Iran University of Medical Sciences, Tehran, Iran

**Keywords:** coronavirus, healthcare professionals, mental health care, mental disorders, psychiatry, psychological distress, public health, SARS-CoV-2

The ongoing COVID-19 pandemic has had far-reaching repercussions on society and individuals' lives, significantly impacting their physical and mental health (1–3). These ramifications have been further influenced by a variety of factors, such as personal circumstances, socioeconomic status, and cultural background (4, 5). With a focus on investigating the effects of the pandemic on mental health, this Research Topic aims to shed light on the role played by sociocultural and personal factors on mental wellbeing. Specifically, the Seventh Volume of our Community Series Research Topic “*Mental Illness, Culture, and Society: Dealing with the COVID-19 Pandemic*” expands upon the findings of the preceding six volumes (6–11) and presents nine articles that explore the impact of the pandemic on the mental health of diverse groups.

Two studies evaluated the mental wellbeing of healthcare workers during the pandemic. Nadeem et al. conducted a cross-sectional study in Pakistan to assess the level of depression, anxiety, and stress among frontline doctors (*n* = 319) and validate the Depression, Anxiety, and Stress Scale (DASS-21). A considerable percentage of respondents had high levels of depression (72.7%), anxiety (70.2%), and stress (58.3%). DASS-21 was validated in the cultural context of Pakistani doctors. Results also revealed a positive correlation between depression and anxiety (r = 0.696, *p* < 0.001), depression and stress (r = 0.761, *p* < 0.001), and anxiety and stress (r = 0.720, *p* < 0.001). In a single group study, Gerbarg et al. evaluated the effects of Breath-Centered Virtual Mind-Body Medicine, the Breath-Body-Mind Introductory Course—BBMIC, on COVID-19-related stress among 39 female healthcare workers in Northern Ireland. Participants completed the Perceived Stress Scale (PSS), Stress Overload Scale-Short (SOS-S), Exercise-Induced Feelings Inventory (EFI), and Indicators of Psychophysiological State (IPSS) at baseline and after finishing the course. Participation in the BBMIC significantly reduced scores on PSS (*p* < 0.011) and EFI subscales for Revitalization (*p* < 0.001), Exhaustion (*p* < 0.002), and Tranquility (*p* < 0.001), but not Engagement. More than 60% reported moderate to very strong improvements in 22 IPSS, including tension, mood, sleep, and mental focus.

Along the same lines, Raeisi et al. assessed the impact of COVID-19 on the children of mothers working as medical staff during the pandemic in Hamadan, Iran. Using a causal-comparative design with a control group, mothers of children aged 6 to 12 years filled information using the Child Behavior Checklist (Achenbach) and the Child Symptom Inventory-4. Compared to controls, children in the staff group scored significantly higher on mean scores of depression, attention problems, and aggression (*p* < 0.05), highlighting the importance of targeted child-parent intervention in this vulnerable group.

Looking at other group populations, Santos et al. assessed the impact of the pandemic on the mental health of Brazilians who reported a positive diagnosis of the disease, with or without symptoms, compared to controls who reported not being diagnosed with COVID-19. Through a cross-sectional design, the authors collected online data from 1,334 people to investigate symptoms of depression, anxiety, post-traumatic stress, and insomnia. The findings highlighted that the pandemic impacted the mental health of individuals regardless of if they were ever diagnosed. Ding et al. investigated the impact of the dynamic zero COVID-19 strategy on pregnant women residing in rural South China. Using a cross-sectional survey, they collected data on anxiety status, sleep quality, physical activity, and diet among 136 pregnant women and 680 controls. Of pregnant women, 25.7, 28.7, and 83.1% had anxiety, sleep problems, and low/medium physical activity, respectively, with no significant difference from the controls. The authors concluded that the strategy had little impact on anxiety, sleep, or physical activity, but affected food intake during pregnancy. Alternatively, in Korea, Lee et al. used an online cross-sectional survey to investigate the association between social determinants of health and perceptions of COVID-19 social distancing, mental health, and quality of life among 1,276 Korean undergraduate students. Compared to those who answered neutrally, students who experienced a negative impact on their social-networking activities due to social distancing were at significantly higher odds to perceive pandemic-related confinement as not being beneficial (OR = 1.948, 95% CI 1.254–3.027) and having elevated stress levels (OR = 1.619, 95% CI 1.051–2.496) and decreased quality of life (OR = 2.230, 95% CI 1.448–3.434). The authors concluded that the social distancing policy may have had a negative impact on the social-networking activities of undergraduate students, emphasizing their need for greater social support and access to resources during periods of confinement.

Using a cross-sectional design, Segura-García et al. also analyzed the impact of social confinement in the first wave of COVID-19 among a group of volunteers in Mexico City. The authors particularly looked at components related to family life, social life, work, mental health, physical activity, and domestic violence. Suffering from domestic violence was significantly associated with having suffered from a symptomatic COVID-19 infection (OR = 4.0099, *p* = 0.0009), being unmarried (OR = 1.4454, *p* = 0.0479), and having poor eating habits (OR = 2.3159, *p* = 0.0084). Despite the policy to assist vulnerable populations during confinement, only a small proportion of the sample reported benefiting from it, emphasizing the importance of improving such policies.

Cohrdes et al. investigated the role of coping factors in maintaining the quality of life among 2,137 German adults during the pandemic, using the Brief COPE and WHOQOL-BREF, respectively. Results of this cross-sectional study showed that participants mostly pursued problem- and meaning-focused coping factors and showed a relatively good quality of life, except for the social domain, with a decreasing trend over time. Escape-avoidance coping was negatively related to all quality-of-life domains, whereas support- and meaning-focused coping showed positive associations (*p* < 0.05). The authors concluded that certain types of coping (support- and meaning-focused) might prevent a decrease in quality of life and should be considered in future health-targeted interventions.

Lastly, in their cross-sectional study, Akingbade et al. looked at the association between electronic health (eHealth) literacy and anxiety and depression during the pandemic in Nigeria. For this purpose, 590 Nigerians filled out the “COVID-19's impAct on feaR and hEalth” (CARE) questionnaire, the eHealth literacy scale, the Patient Health Questionnaire-4, and the COVID-19 fear scale. High eHealth literacy was associated with lower odds of anxiety (aOR = 0.34, 95% CI 0.20–0.54) and depression (aOR = 0.34, 95% CI 0.21–0.56). There were age, sex, and regional differences in the associations between eHealth literacy and psychological outcomes. The authors emphasized the importance of digital health information to improve access and delivery of mental health services.

In conclusion, the papers compiled in the Seventh Volume of this Research Topic offer an insightful outlook on the effects of COVID-19 on mental health, further highlighting the intricate interplay among sociocultural, economic, and individual factors. It is necessary to acknowledge that the impact of the pandemic, whether on mental wellbeing or society, extends beyond a temporary disruption, necessitating continued attention and comprehensive understanding. To address the specific vulnerabilities faced by different groups, there is a crucial need for further clinical and epidemiological research, as well as the provision of appropriately tailored resources and interventions.

## Author contributions

All authors listed have made a substantial, direct, and intellectual contribution to the work and approved it for publication.
